# Supplementary material for: Depletion of mitochondrial methionine adenosyltransferase α1 triggers mitochondrial dysfunction in alcohol-associated liver disease
Source: Nat Commun. 2022 Jan 28;13:557. doi: 10.1038/s41467-022-28201-2 (PMC8799735; doi:10.1038/s41467-022-28201-2)

Supplementary information for:

Depletion of Mitochondrial Methionine Adenosyltransferase α1 Triggers Mitochondrial Dysfunction in Alcohol-Associated Liver Disease

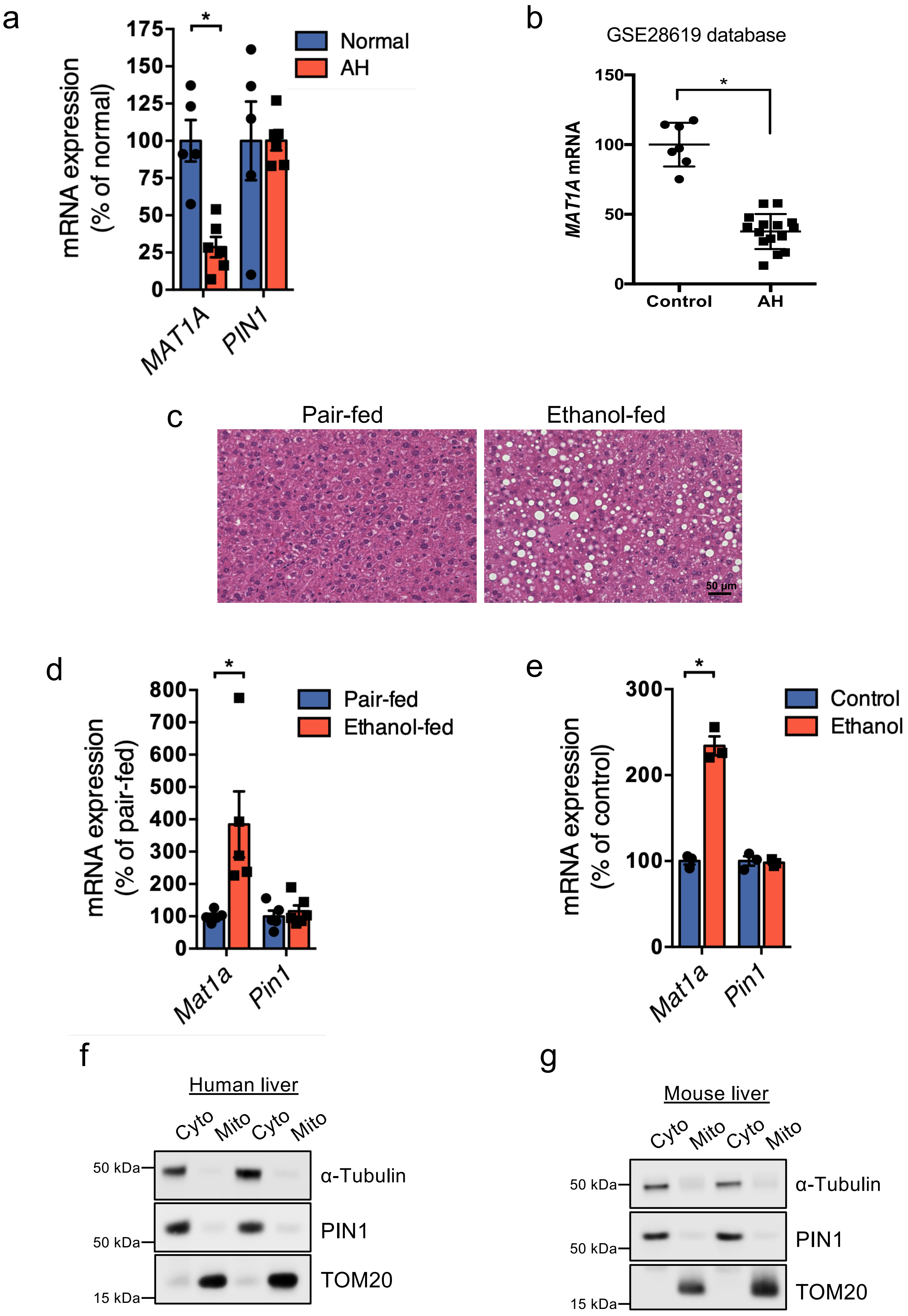

Suppl. Figure 1

Supplemental figure 1. *MAT1A* and *PIN1* mRNA levels and purity of fractions. (a) *MAT1A* and *PIN1* mRNA levels in normal (n=5) and AH (n=6) human liver samples (*MAT1A* p=0.0009). (b) *MAT1A* mRNA levels in control (n=7) and AH (n=15) human livers from the public database GSE28619 (p=2.0E-09). (c) Hematoxylin & Eosin staining in livers of pair-fed and ethanol-fed mice following the NIAAA diet. *Mat1a* and *Pin1* mRNA levels in (d) pair-fed (n=5) and ethanol-fed (n=5) mouse livers (*Mat1a* p=0.02) and (e) AML-12 cells treated with ethanol 100 mM for 48 hours (*Mat1a* p=0.0003, n=3 independent experiments). (f-g) Western blots against PIN1, α-Tubulin, TOM20 in the cytosolic and mitochondrial fractions of (f) human livers and (g) mouse livers. *p<0.05. Statistical significance was determined by using two-tailed, one-sample t-test. Data are presented as mean values ± SEM. Source data are provided as a Source Data file. Controls are shown in blue and AH, ethanol-fed and ethanol are shown in red. AH: alcoholic hepatitis.

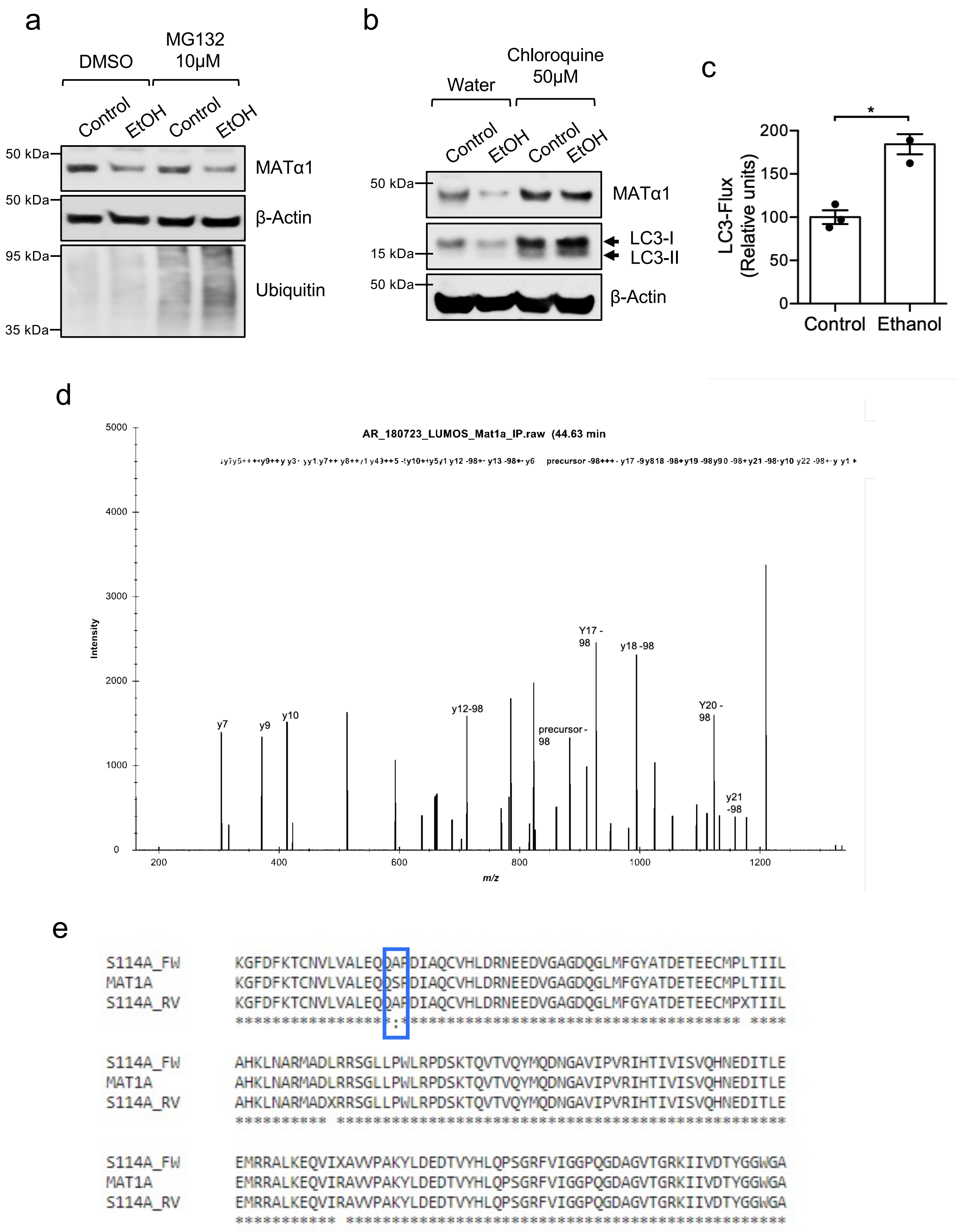

Suppl. Figure 2

Supplemental figure 2. Ethanol promotes MATα1 degradation through autophagy. (a) Western blot of MATα1 in AML-12 cells treated with ethanol and the proteasome inhibitor MG132 (n= 3 independent experiments). (b) Western blots of MATα1 and LC3 in AML-12 cells treated with ethanol and the autophagy inhibitor chloroquine (CQ) (n= 3 independent experiments). (c) Autophagic flux calculated as the accumulation of LC3-II after the addition of CQ in AML-12 cells treated with ethanol (n= 3 independent experiments, p=0.004). (d) Representative spectra obtained from DDA-MS acquisitions in MATα1 immunoprecipitations showing all of the ions used to identify the peptide containing MATα1 Ser114. (e) MATα1 WT and S114A sequence alignment showing serine114 mutation to alanine. *$p<0.05$. Statistical significance was determined by using two-tailed, one-sample t-test. Data are presented as mean values ± SEM. Source data are provided as a Source Data file.

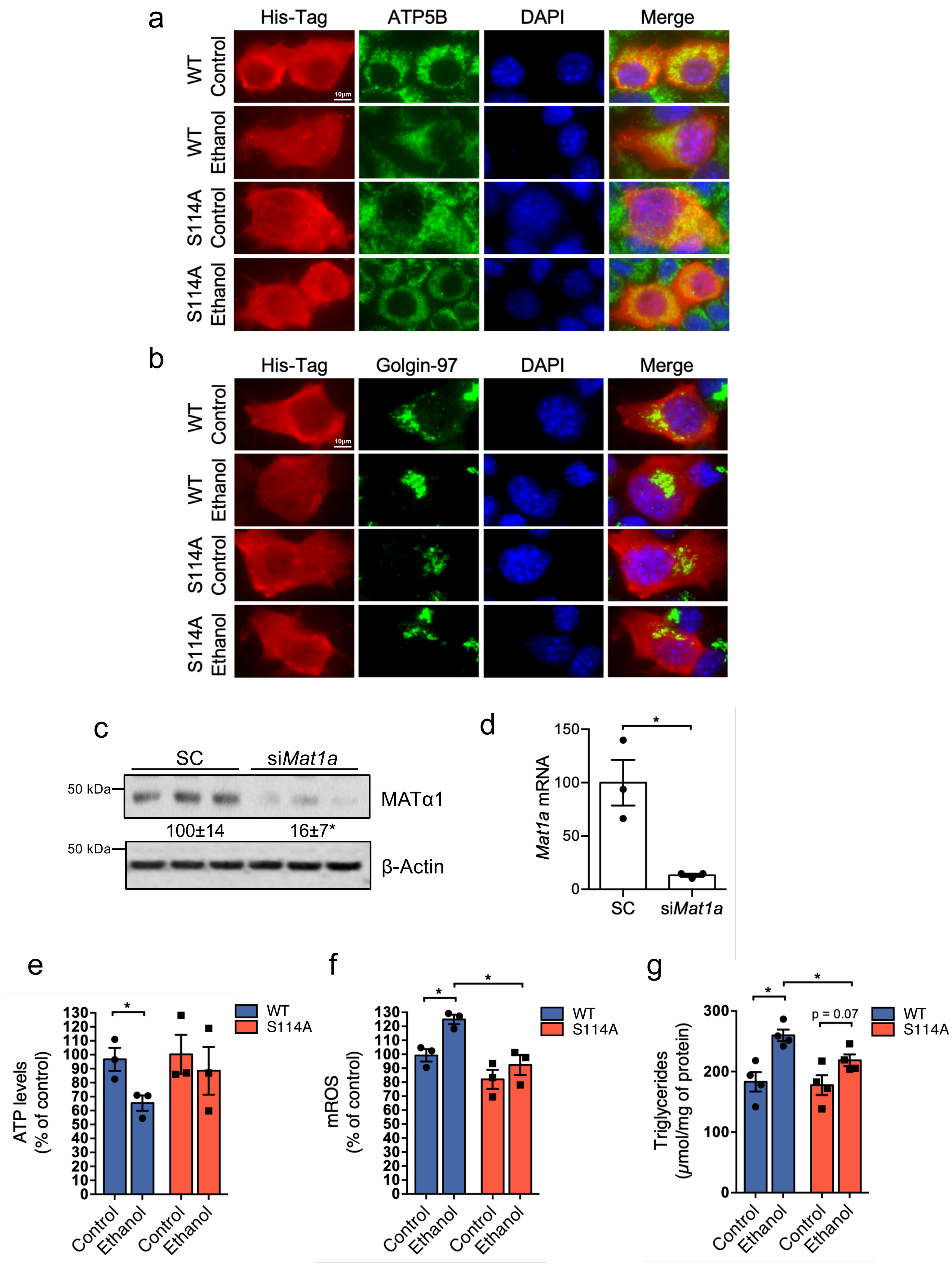

Suppl. Figure 3

Supplemental figure 3. Blocking PIN1-MATα1 interaction protects against alcohol-induced injury by increasing MATα1 mitochondrial content. (a) His-Tag (red) and ATP synthase (green) and (b) His-Tag (red) and Golgin-97 (green) immunofluorescence in AML-12 cells that were overexpressed with MATα1 WT or S114A in His-Tag and treated with ethanol. (c) Western blot of MATα1 and (d) *Mat1a* mRNA levels in AML-12 after *Mat1a* silencing (p=0.016). (e) ATP (p=0.034 WT ethanol vs control), (f) mROS levels (p=0.010 WT ethanol vs control; p=0.015 S114A ethanol vs WT ethanol), and (g) triglycerides content (n=4 independent experiments, p=0.034 WT ethanol vs control; p=0.025 S114A ethanol vs WT ethanol), in AML-12 cells expressing MATα1 WT or S114A after *Mat1a* silencing and ethanol treatment. n= 3 independent experiments unless specified. *p<0.05. Statistical significance was determined by using two-tailed, one-sample t-test for treatment comparisons and ANOVA test for group comparisons. Data are presented as mean values ± SEM. Data are presented as mean values ± SEM. Source data are provided as a Source Data file. WT is shown in blue and S114A in red. mROS: mitochondrial reactive oxygen species; SC: scramble; si: silencing; WT: wild type.

a

Program	Kinase	Score
Netphos 3.1	p38	0.516
	GSK3	0.471
	CDC2	0.465
	CaM-II	0.459
	CK2	0.396
NetPhorest	p38	0.15
	JNK	0.13
	CDK1/2/3/5	0.04
	CaM-II	0.03
	CK2	0.02
GSP 3.0	p38	121.73
	GSK3B	8.08
	JNK	3.7
	CK2	2.71
	CaM-II	1.8

b

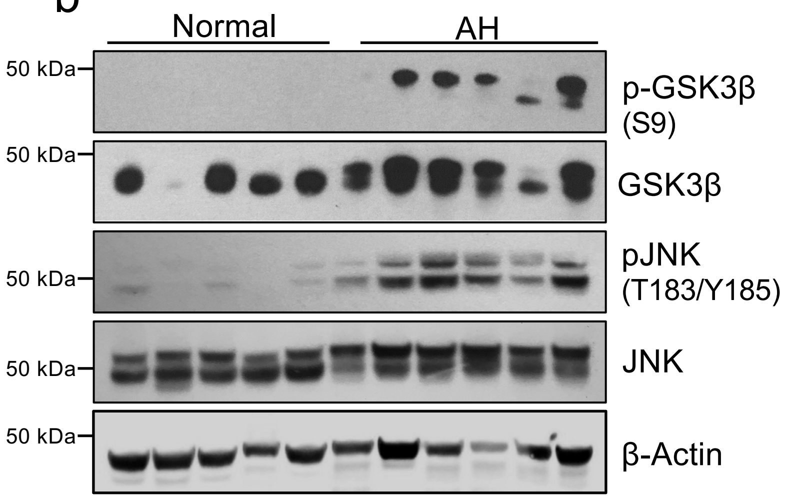

c

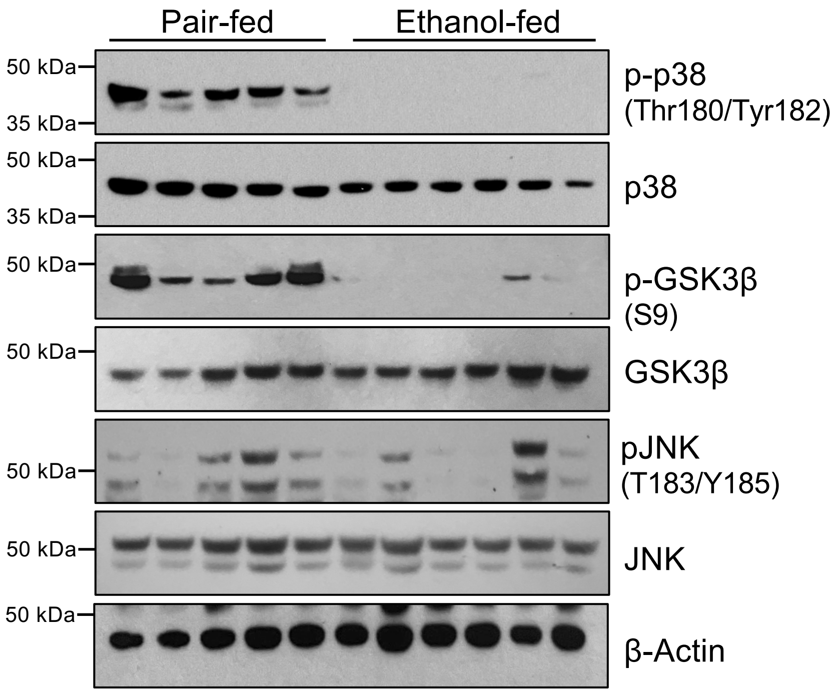

d

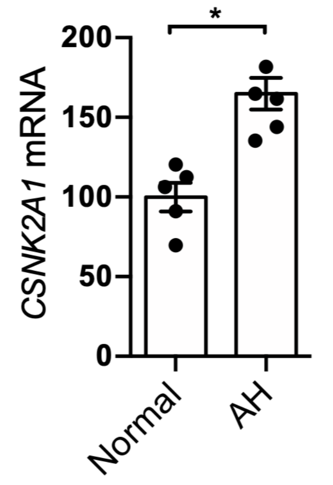

e

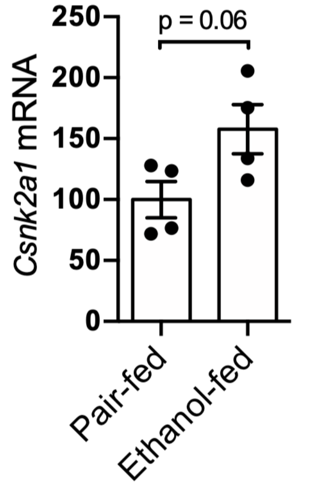

f

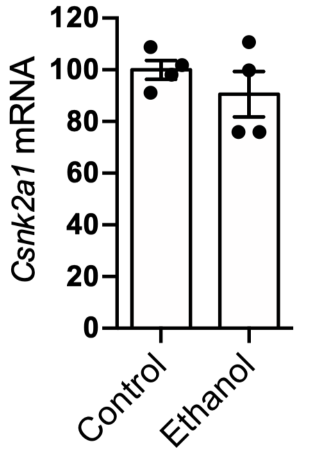

g

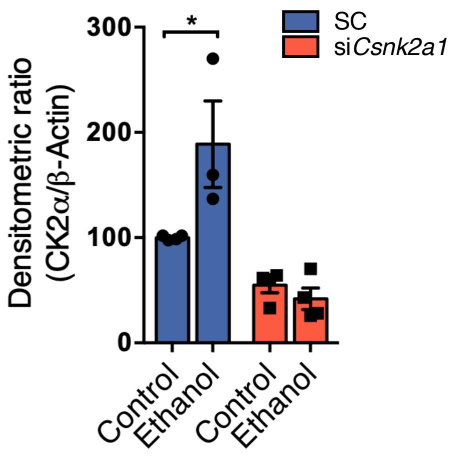

Suppl. Figure 4

Supplemental figure 4. Potential kinases that phosphorylate MATα1 Ser114 in ALD. (a) Kinase predictions for Ser114 phosphorylation. Western blots of shown kinases in (b) human normal (n=5) and AH (n=6) livers and (c) pair-fed (n=5) and ethanol-fed (n=6) mouse livers. *CSNK2A1* mRNA levels in (d) human normal (n=5) and AH livers(n=6) (p=0.0009), (e) pair-fed (n=4) and ethanol-fed (n=4) mouse livers and (f) AML-12 cells treated with ethanol (n=4 independent experiments). (g) Densitometry analyses of CK2α in AML-12 cells treated with ethanol after *Csnk2a1* silencing (p=0.034 SC ethanol vs SC control). *p<0.05. Statistical analyses were performed using two-tail Fisher's test for treatment comparisons. Data are presented as mean values ± SEM. Source data are provided as a Source Data file. AH: alcoholic hepatitis; SC: scramble; si: silencing.

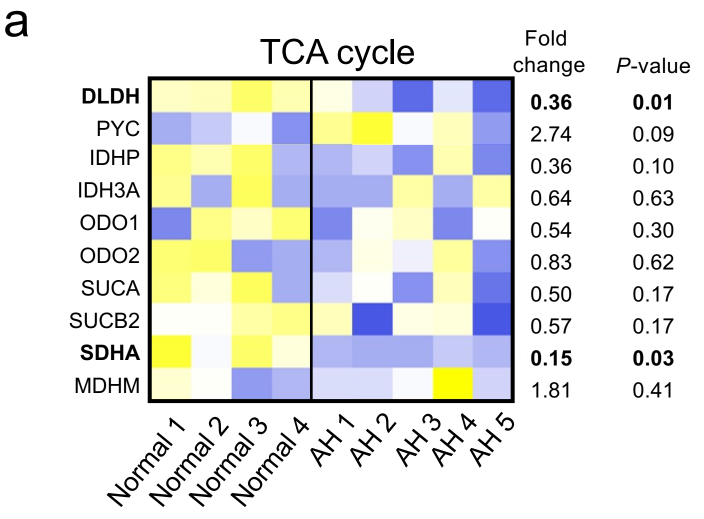

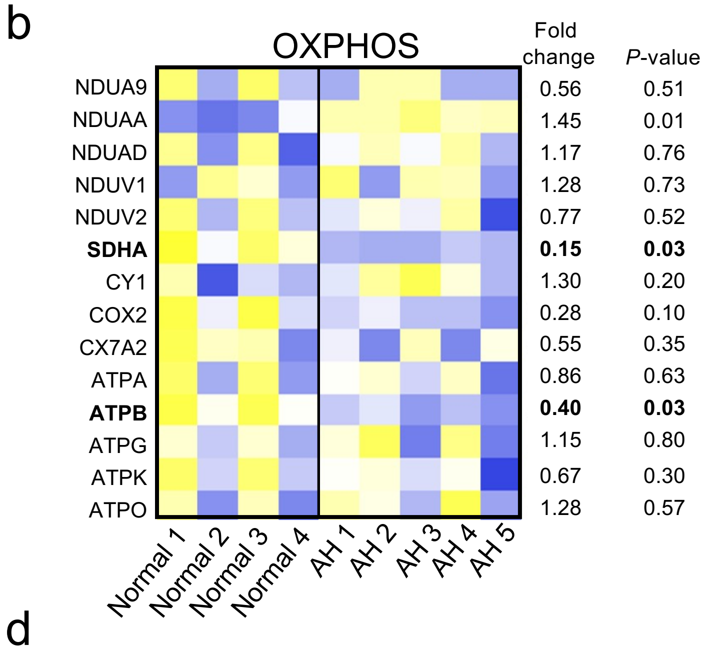

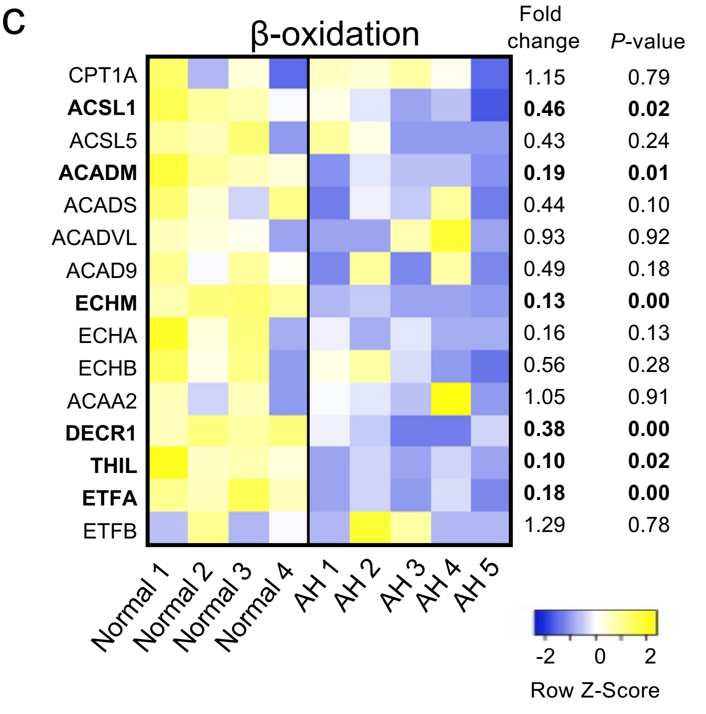

d

**KEGG pathway**	**Count**	**%**	**FDR**
Metabolic pathways	66	50	<0.001
Carbon metabolism	21	15.9	<0.001
Fatty acid degradation	13	9.8	<0.001
Fatty acid metabolism	10	7.6	<0.001
Glycolysis / Gluconeogenesis	8	6.1	<0.001
Citrate cycle (TCA cycle)	6	4.5	<0.001
Synthesis and degradation of ketone bodies	4	3	<0.001
Pyruvate metabolism	5	3.8	0.002
Oxidative phosphorylation	4	3	0.034

Suppl. Figure 5

Supplemental Figure 5. MATα1 interaction with mitochondrial enzymes of the TCA cycle, OXPHOS, and fatty acid β-oxidation is reduced in ALD. (a-c) Heatmaps with the intensities (converted into Z-scores) of the mitochondrial proteins involved in (a) TCA cycle, (b), OXPHOS and (c) β-oxidation detected in normal (n=4) and AH (n=5) human livers after MATα1 co-IP and identified by DIA-MS. Score ranges from lower (blue) to higher (yellow) (n=4 independent experiments). (d) Pathways predicted to be regulated in AH based on the proteins having reduced interaction with MATα1 by the DAVID online software. Significant values ($p<0.05$) are shown in bold. Statistical significance was determined by using two-tailed, one-sample t-test. FDR: false discovery rate; KEGG: Kyoto Encyclopedia of Genes and Genomes OXPHOS: oxidative phosphorylation; TCA: tricarboxylic.

Supplemental table 1. Functional analysis of proteins found to interact less with MATα1 (n=132) (see supplemental file 3) using DAVID online database. Statistical analyses were performed by using one-sided Fisher's exact test.

GO Term	Count	%	P-Value
Oxidation-reduction process	28	21.2	<0.001
Fatty acid β-oxidation	10	7.6	<0.001
Citrate cycle (TCA cycle)	7	5.3	<0.001
Ketone body biosynthetic process	4	3	<0.001
Glycolytic process	4	3	0.002
Succinate metabolic process	3	2.3	0.003
Lipid metabolic process	6	4.5	0.007
Response to fatty acid	3	2.3	0.007
Fatty acid metabolic process	4	3	0.007
2-oxoglutarate metabolic process	3	2.3	0.008
Fatty acid beta-oxidation using acyl-CoA dehydrogenase	3	2.3	0.008
Respiratory electron transport chain	3	2.3	0.010
Carbohydrate metabolic process	6	4.5	0.011
Pyruvate metabolic process	3	2.3	0.012
ATP biosynthetic process	3	2.3	0.021
Mitochondrion organization	4	3	0.021
Medium-chain fatty acid metabolic process	2	1.5	0.023
Triglyceride metabolic process	3	2.3	0.030
Lipid homeostasis	3	2.3	0.036
Carnitine biosynthetic process	2	1.5	0.038
Succinyl-CoA metabolic process	2	1.5	0.038
Gluconeogenesis	3	2.3	0.045

Count: number of proteins with lower interaction with MATα1 associated with that GO term.

%: percentage of proteins with lower interaction with MATα1 associated with that GO term.

Supplemental table 2. Pathway analysis of proteins found to interact more with MATα1 (n=202) (see supplemental file 3) using DAVID online database. Statistical analyses were performed by using one-sided Fisher's exact test.

**KEGG pathway**	**Count**	**%**	**P-Value**
Ribosome	26	12.9	<0.001
Protein processing in endoplasmic reticulum	12	5.9	<0.001
Primary bile acid biosynthesis	4	2	0.005
Selenocompound metabolism	3	1.5	0.049
Aminoacyl-tRNA biosynthesis	5	2.5	0.051
Proteasome	4	2	0.066

Count: number of proteins with higher interaction with MATα1 associated with that KEGG pathway.

%: percentage of proteins with higher interaction with MATα1 associated with that KEGG pathway

Original gels for Supplementary Figure 1

Fig. S1f)

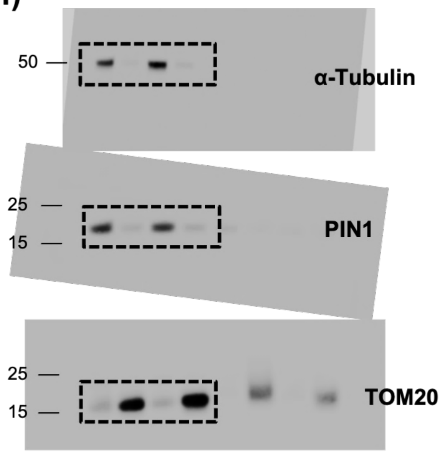

Fig. S1g)

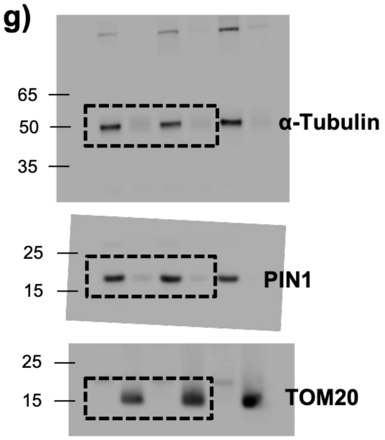

Original gels for Supplementary Figure 2

Fig. S2a)

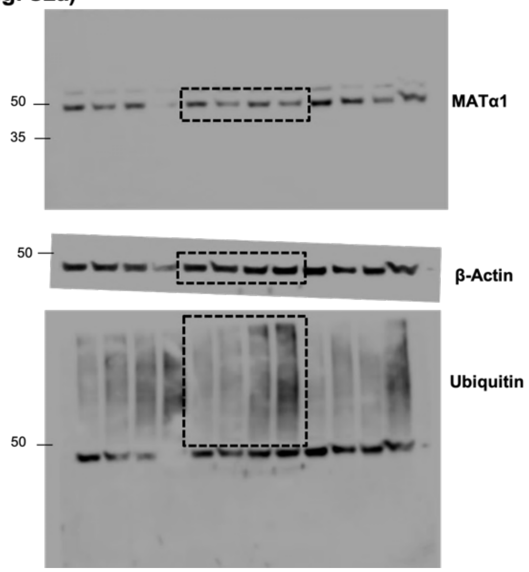

Fig. S2b)

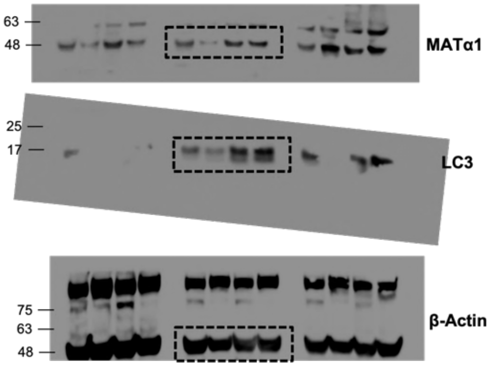

Original gels for Supplementary Figure 3

Fig. S3c)

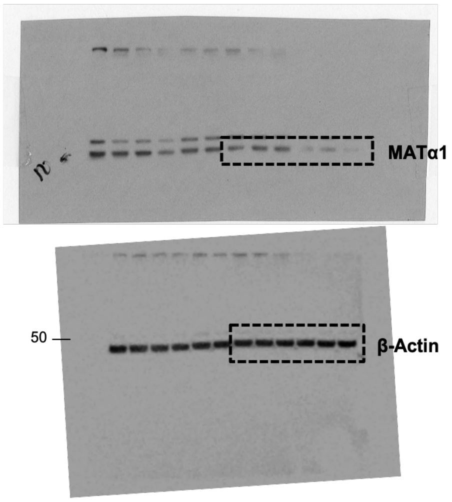

Original gels for Supplementary Figure 4

Fig. S4b)

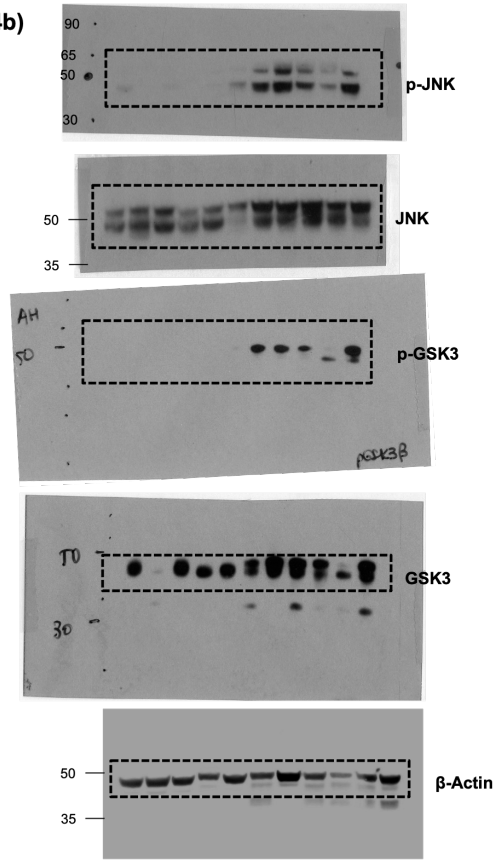

Fig. S4c)

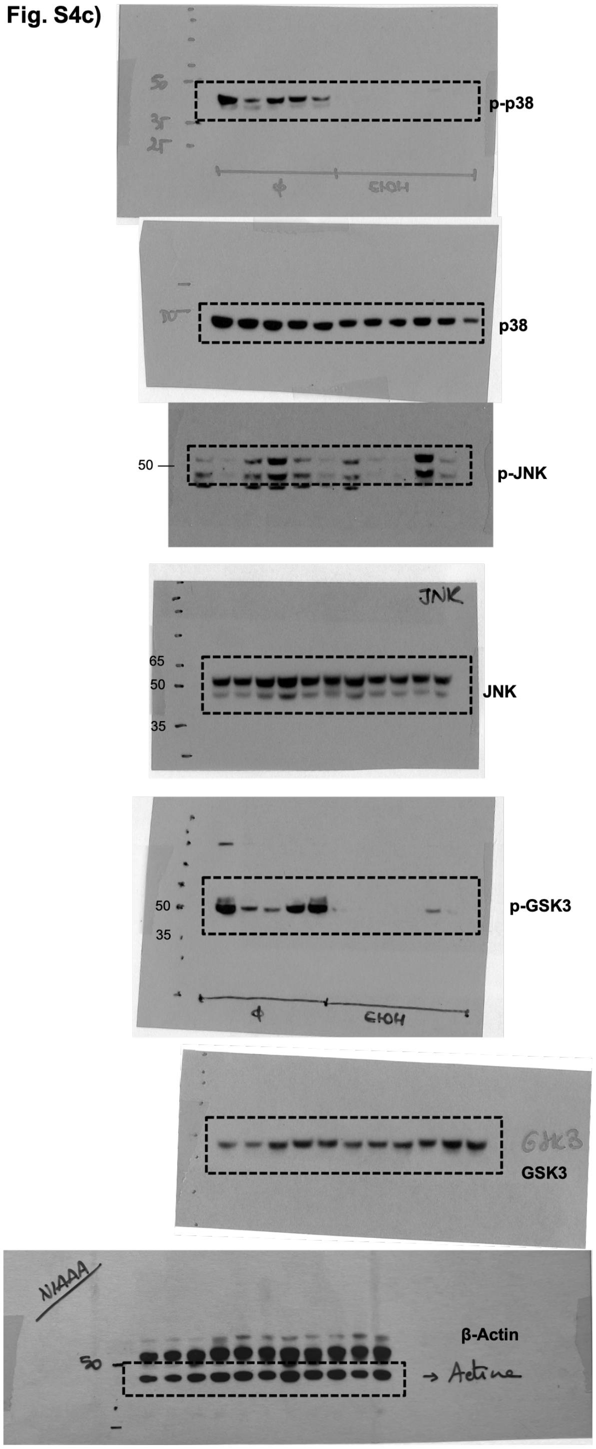

Supplement: Supplementary file 1 — Supplementary Information [file 41467_2022_28201_MOESM1_ESM.pdf]
