# Supplementary material for: Depletion of mitochondrial methionine adenosyltransferase α1 triggers mitochondrial dysfunction in alcohol-associated liver disease
Source: Nat Commun. 2022 Jan 28;13:557. doi: 10.1038/s41467-022-28201-2 (PMC8799735; doi:10.1038/s41467-022-28201-2)
Supplement: Supplementary file 3 — Description of Additional Supplementary Files [file 41467_2022_28201_MOESM3_ESM.pdf]

<u>Inventory of Supporting Information</u>

File name: Supplementary information file
Description: Supplementary figure and figure legends, supplementary tables 1 and 2 and original gels for Supplementary Figures.

File name: Supplementary data 1
Description: Ser114 phosphorylation analyses in normal and AH human livers.

File name: Supplementary data 2
Description: List of identified mitochondrial proteins in normal human livers after MATα1 co-IP+MS analyses.

File name: Supplementary data 3
Description: Quantification of identified mitochondrial proteins in normal and AH human livers after MATα1 co-IP+MS analyses.

File name: Supplementary data 4
Description: Mitoplex raw data.
