# Supplementary material for: Depletion of mitochondrial methionine adenosyltransferase α1 triggers mitochondrial dysfunction in alcohol-associated liver disease
Source: Nat Commun. 2022 Jan 28;13:557. doi: 10.1038/s41467-022-28201-2 (PMC8799735; doi:10.1038/s41467-022-28201-2)
Supplement: Supplementary file 8 — Reporting Summary [file 41467_2022_28201_MOESM8_ESM.pdf]

nature research

Corresponding author(s): Shelly C. Lu

Last updated by author(s): 2021-12-08

Reporting Summary

Nature Research wishes to improve the reproducibility of the work that we publish. This form provides structure for consistency and transparency in reporting. For further information on Nature Research policies, see our Editorial Policies and the Editorial Policy Checklist.

Statistics

For all statistical analyses, confirm that the following items are present in the figure legend, table legend, main text, or Methods section.

n/a	Confirmed	
☐	☑	The exact sample size (n) for each experimental group/condition, given as a discrete number and unit of measurement
☐	☑	A statement on whether measurements were taken from distinct samples or whether the same sample was measured repeatedly
☐	☑	The statistical test(s) used AND whether they are one- or two-sided<br>*Only common tests should be described solely by name; describe more complex techniques in the Methods section.*
☐	☑	A description of all covariates tested
☑	☐	A description of any assumptions or corrections, such as tests of normality and adjustment for multiple comparisons
☐	☑	A full description of the statistical parameters including central tendency (e.g. means) or other basic estimates (e.g. regression coefficient) AND variation (e.g. standard deviation) or associated estimates of uncertainty (e.g. confidence intervals)
☐	☑	For null hypothesis testing, the test statistic (e.g. F, t, r) with confidence intervals, effect sizes, degrees of freedom and P value noted<br>*Give P values as exact values whenever suitable.*
☑	☐	For Bayesian analysis, information on the choice of priors and Markov chain Monte Carlo settings
☑	☐	For hierarchical and complex designs, identification of the appropriate level for tests and full reporting of outcomes
☑	☐	Estimates of effect sizes (e.g. Cohen's d, Pearson's r), indicating how they were calculated

Our web collection on statistics for biologists contains articles on many of the points above.

Software and code

Policy information about availability of computer code

Data collection

Western blots were obtained by regular film developing and the digital chemoluminescent detector Azure 280. Immunofluorescence and oil red o images were captured on a Keyence microscope (BZ-X710). Absorbance, luminescence and fluorescence assays were measured with a ClarioSTAR (BMG Labtech) microplate reader. Respiratory analyses were performed using a Seahorse XFe90 Analyzer. MS data was collected with an Orbitrap LUMOS Fusion mass spectrometer and Prominence UFLCXR HPLC system. NRM was measured on a Bruker 600 MHz (12 T) Avance III spectrometer.

Data analysis

Image J 1.52q was used to perform densitometry analyses and staining quantifications. All data analysis was finalized usinf Excel (16.54) and Graphpad Prism 9.0.0. David 6.8 was used to analyze GO terms and KEGG pathways. STRING 11.0 was used to analyze GO terms and KEGG pathways. Skyline version 19.1.1.309 was used to analyze (visualize, select peak boundaries and quantify the area under the curve) mass spectrometry raw data. PyProphet version 2.1.2 was used to analyze target and decoy peptides.

For manuscripts utilizing custom algorithms or software that are central to the research but not yet described in published literature, software must be made available to editors and reviewers. We strongly encourage code deposition in a community repository (e.g. GitHub). See the Nature Research guidelines for submitting code & software for further information.

Data

Policy information about availability of data

All manuscripts must include a data availability statement. This statement should provide the following information, where applicable:

- Accession codes, unique identifiers, or web links for publicly available datasets
- A list of figures that have associated raw data
- A description of any restrictions on data availability

Raw data from each figure or table are provided as a Source Data file and Supplementary files. Source data are provided with this paper. All the mass spectrometry proteomics data have been deposited to the ProteomeXchange Consortium via the PRIDE [1] partner repository with the dataset identifier PXD030383. Data in Figure S1b is from publicly available database (GSE28619). DAVID and STRING are publicly available databases.

Field-specific reporting

Please select the one below that is the best fit for your research. If you are not sure, read the appropriate sections before making your selection.

☒ Life sciences ☐ Behavioural & social sciences ☐ Ecological, evolutionary & environmental sciences

For a reference copy of the document with all sections, see nature.com/documents/nr-reporting-summary-flat.pdf

Life sciences study design

All studies must disclose on these points even when the disclosure is negative.

Sample size	*A reasonable sample size was estimated from similar experiments in others' and our former publications to ensure adequate reproducibility of results. The exact n values used to calculate the statistics are provided.*
Data exclusions	*No data was excluded from the analysis.*
Replication	*All experiments were performed independently 3 times unless stated differently in the figure legend. All attempts at replication were successful under the experimental conditions defined.*
Randomization	*Animals with similar age and weight were randomly allocated to experimental groups. For in vitro experiments, cultures were randomly chosen for different treatments and experiments were performed multiple times.*
Blinding	*Investigators were blinded to group allocation during data collection and/or analysis.*

Behavioural & social sciences study design

All studies must disclose on these points even when the disclosure is negative.

Study description	*Briefly describe the study type including whether data are quantitative, qualitative, or mixed-methods (e.g. qualitative cross-sectional, quantitative experimental, mixed-methods case study).*
Research sample	*State the research sample (e.g. Harvard university undergraduates, villagers in rural India) and provide relevant demographic information (e.g. age, sex) and indicate whether the sample is representative. Provide a rationale for the study sample chosen. For studies involving existing datasets, please describe the dataset and source.*
Sampling strategy	*Describe the sampling procedure (e.g. random, snowball, stratified, convenience). Describe the statistical methods that were used to predetermine sample size OR if no sample-size calculation was performed, describe how sample sizes were chosen and provide a rationale for why these sample sizes are sufficient. For qualitative data, please indicate whether data saturation was considered, and what criteria were used to decide that no further sampling was needed.*
Data collection	*Provide details about the data collection procedure, including the instruments or devices used to record the data (e.g. pen and paper, computer, eye tracker, video or audio equipment) whether anyone was present besides the participant(s) and the researcher, and whether the researcher was blind to experimental condition and/or the study hypothesis during data collection.*
Timing	*Indicate the start and stop dates of data collection. If there is a gap between collection periods, state the dates for each sample cohort.*
Data exclusions	*If no data were excluded from the analyses, state so OR if data were excluded, provide the exact number of exclusions and the rationale behind them, indicating whether exclusion criteria were pre-established.*
Non-participation	*State how many participants dropped out/declined participation and the reason(s) given OR provide response rate OR state that no participants dropped out/declined participation.*
Randomization	*If participants were not allocated into experimental groups, state so OR describe how participants were allocated to groups, and if allocation was not random, describe how covariates were controlled.*

Ecological, evolutionary & environmental sciences study design

All studies must disclose on these points even when the disclosure is negative.

Study description	*Briefly describe the study. For quantitative data include treatment factors and interactions, design structure (e.g. factorial, nested, hierarchical), nature and number of experimental units and replicates.*
Research sample	*Describe the research sample (e.g. a group of tagged Passer domesticus, all Stenocereus thurberi within Organ Pipe Cactus National*

Research sample	*Monument), and provide a rationale for the sample choice. When relevant, describe the organism taxa, source, sex, age range and any manipulations. State what population the sample is meant to represent when applicable. For studies involving existing datasets, describe the data and its source.*
Sampling strategy	*Note the sampling procedure. Describe the statistical methods that were used to predetermine sample size OR if no sample-size calculation was performed, describe how sample sizes were chosen and provide a rationale for why these sample sizes are sufficient.*
Data collection	*Describe the data collection procedure, including who recorded the data and how.*
Timing and spatial scale	*Indicate the start and stop dates of data collection, noting the frequency and periodicity of sampling and providing a rationale for these choices. If there is a gap between collection periods, state the dates for each sample cohort. Specify the spatial scale from which the data are taken*
Data exclusions	*If no data were excluded from the analyses, state so OR if data were excluded, describe the exclusions and the rationale behind them, indicating whether exclusion criteria were pre-established.*
Reproducibility	*Describe the measures taken to verify the reproducibility of experimental findings. For each experiment, note whether any attempts to repeat the experiment failed OR state that all attempts to repeat the experiment were successful.*
Randomization	*Describe how samples/organisms/participants were allocated into groups. If allocation was not random, describe how covariates were controlled. If this is not relevant to your study, explain why.*
Blinding	*Describe the extent of blinding used during data acquisition and analysis. If blinding was not possible, describe why OR explain why blinding was not relevant to your study.*

Did the study involve field work? ☐ Yes ☐ No

Field work, collection and transport

Field conditions	*Describe the study conditions for field work, providing relevant parameters (e.g. temperature, rainfall).*
Location	*State the location of the sampling or experiment, providing relevant parameters (e.g. latitude and longitude, elevation, water depth).*
Access & import/export	*Describe the efforts you have made to access habitats and to collect and import/export your samples in a responsible manner and in compliance with local, national and international laws, noting any permits that were obtained (give the name of the issuing authority, the date of issue, and any identifying information).*
Disturbance	*Describe any disturbance caused by the study and how it was minimized.*

Reporting for specific materials, systems and methods

We require information from authors about some types of materials, experimental systems and methods used in many studies. Here, indicate whether each material, system or method listed is relevant to your study. If you are not sure if a list item applies to your research, read the appropriate section before selecting a response.

Materials & experimental systems

n/a	Involved in the study	
☐	☑	Antibodies
☐	☑	Eukaryotic cell lines
☑	☐	Palaeontology and archaeology
☐	☑	Animals and other organisms
☐	☑	Human research participants
☑	☐	Clinical data
☑	☐	Dual use research of concern

Methods

n/a	Involved in the study	
☑	☐	ChIP-seq
☑	☐	Flow cytometry
☑	☐	MRI-based neuroimaging

Antibodies

Antibodies used	*ATP Synthase β (Abcam, ab128743, IF 1/100), β-Actin (Sigma, A3854, AC-15, WB 1/5000), CK2α (Cell Signaling, 2656, WB 1/1000), CPT1α (Cell Signaling, 12252, D3B3, WB 1/1000), DDK (Origene, TA50011-100, OTI4C5, WB 1/1000), GAPDH (Cell Signaling, 5174, D16H11, WB 1/1000), Golgin-97 (Proteintech, 12640-1-AP, IF 1/100), GSK3β (Cell Signaling, 9315, 27C10, WB 1/1000), p-GSK3β (Cell Signaling, 9336, WB 1/1000), His-Tag (GenScript, A00186-100, 6G2A9, WB 1/1000, IP 3 μg), His-Tag (Proteintech, 66005-1-Ig, 1B7G5, IF 1/100), JNK (Cell Signaling, 9252, WB 1/1000), p-JNK (Cell Signaling, 4668, 81E11, WB 1/1000), LC3 (Abcam, ab128025, WB 1/1000), MATα1 (Abcam, ab129176, EPR7938, WB 1/1000, IP 3 μg), MCAD (Abcam, ab110296, 3B7BH7, WB 1/1000), Methyl-lysine (Abcam, ab23366, WB 1/1000), OXPHOS (Abcam, ab110413, WB 1/1000), p38 (Cell Signaling, 9212, WB 1/1000), p-p38 (Cell Signaling, 9211, WB 1/1000), PIN1 (Millipore, 07-091, WB 1/1000, IP 3 μg), Phospho-serine (Abcam, ab9332, IP 3 μg), SDHα (Cell Signaling, 5839, WB 1/1000), TOM20 (Abcam, ab78547, WB 1/1000), TOM70 (Proteintech, 14528-1-AP, IF 1/100), α-Tubulin (Invitrogen, 32-25002, B-5-1-2, WB 1/1000), Ubiquitin (Proteintech, 10201-2-AP, WB 1/500), anti-rabbit (Cell Signaling, 7074S, WB 1/5000), anti-mouse (Cell Signaling, 7076S, WB 1/5000), Clean Blot (Thermo Fisher, 21230, WB 1/500-1/2000), Alexa fluor 488 (Abcam, ab150077, IF 1/200), Cy3 (Jackson ImmunoResearch, 115-165-003, IF 1/200).*
Validation	*ATP Synthase β (Abcam, ab128743, https://www.abcam.com/atpb-antibody-mitochondrial-marker-ab128743.html?productWallTab=Abreviews); β-Actin (Sigma, A3854, AC-15, https://www.sigmaaldrich.com/US/en/product/sigma/a3854); CK2α (Cell Signaling, 2656, https://www.cellsignal.com/products/primary-antibodies/ck2a-antibody/2656. Validated by Csnk2a1 silencing); CPT1α (Cell Signaling, 12252, https://www.cellsignal.com/products/primary-antibodies/cpt1a-d3b3-rabbit-mab/12252); DDK (Origene, TA50011-100, OTI4C5, https://www.thermofisher.com/antibody/product/DDK-Antibody-clone-OTI4C5-Monoclonal/TA50011-100. Validated with DDK-tagged protein); GAPDH (Cell Signaling, 5174, D16H11, https://www.cellsignal.com/products/primary-antibodies/gapdh-d16h11-xp-rabbit-mab/5174); Golgin-97 (Proteintech, 12640-1-AP, https://www.ptglab.com/products/GOLGA1-Antibody-12640-1-AP.htm); GSK3β (Cell Signaling, 9315, 27C10, https://www.cellsignal.com/products/primary-antibodies/gsk-3b-27c10-rabbit-mab/9315); p-GSK3β (Cell Signaling, 9336, https://www.cellsignal.com/products/primary-antibodies/phospho-gsk-3b-ser9-antibody/9336); His-Tag (GenScript, A00186-100, 6G2A9, https://www.genscript.com/antibody/A00186_100-THE_sup_TM_sup_His_Tag_Antibody_mAb_Mouse.html?src=biocompare. Validated with His-tagged protein); His-Tag (Proteintech, 66005-1-Ig, 1B7G5, https://www.ptglab.com/products/His-Tag-Antibody-66005-1-Ig.htm); JNK (Cell Signaling, 9252, https://www.cellsignal.com/products/primary-antibodies/sapk-jnk-antibody/9252); p-JNK (Cell Signaling, 4668, 81E11, https://www.cellsignal.com/products/primary-antibodies/phospho-sapk-jnk-thr183-tyr185-81e11-rabbit-mab/4668); LC3 (Abcam, ab128025, https://www.abcam.com/lc3ab-antibody-ab128025.html); MATα1 (Abcam, ab129176, EPR7938, https://www.abcam.com/mat1a-antibody-epr7938-ab129176.html. Validated in Mat1a-KO mice); MCAD (Abcam, ab110296, 3B7BH7, https://www.abcam.com/acadmmcad-antibody-3b7bh7-ab110296.html); Methyl-lysine (Abcam, ab23366, https://www.abcam.com/methylated-lysine-di-methyl--mono-methyl--antibody-ab23366.html); OXPHOS (Abcam, ab110413, https://www.abcam.com/total-oxphos-rodent-wb-antibody-cocktail-ab110413.html); p38 (Cell Signaling, 9212, https://www.cellsignal.com/products/primary-antibodies/p38-mapk-antibody/9212); p-p38 (Cell Signaling, 9211, https://www.cellsignal.com/products/primary-antibodies/phospho-p38-mapk-thr180-tyr182-antibody/9211); PIN1 (Millipore, 07-091, https://www.emdmillipore.com/US/en/product/Anti-Pin1-Antibody,MM_NF-07-091?ReferrerURL=https%3A%2F%2Fwww.google.com%2F. Validated by Pin1 silencing and in Pin1-KO mice); Phospho-serine (Abcam, ab9332, https://www.abcam.com/phosphoserine-antibody-ab9332.html); SDHα (Cell Signaling, 5839, https://www.cellsignal.com/products/primary-antibodies/sdha-antibody/5839); TOM20 (Abcam, ab78547, https://www.abcam.com/tomm20-antibody-mitochondrial-marker-ab78547.html); TOM70 (Proteintech, 14528-1-AP, https://www.ptglab.com/products/TOM70-Antibody-14528-1-AP.htm); α-Tubulin (Invitrogen, 32-25002, B-5-1-2, https://www.thermofisher.com/antibody/product/alpha-Tubulin-Antibody-clone-B-5-1-2-Monoclonal/32-2500); Ubiquitin (Proteintech, 10201-2-AP, https://www.ptglab.com/products/ubiquitin-Antibody-10201-2-AP.htm); anti-rabbit (Cell Signaling, 7074S, https://www.cellsignal.com/products/secondary-antibodies/anti-rabbit-igg-hrp-linked-antibody/7074); anti-mouse (Cell Signaling, 7076S, https://www.cellsignal.com/products/secondary-antibodies/anti-mouse-igg-hrp-linked-antibody/7076); Alexa fluor 488 (Abcam, ab150077, https://www.abcam.com/goat-rabbit-igg-hl-alexa-fluor-488-ab150077.html); Cy3 (Jackson ImmunoResearch, 115-165-003, https://www.jacksonimmuno.com/catalog/products/115-165-003).*

Eukaryotic cell lines

Policy information about cell lines

Cell line source(s)	*Murine AML-12 and human HepG2 cells were obtained from American Type Culture Collection (Rockville, MD).*
Authentication	*No authentication has been conducted for AML-12 after purchase. HepG2 were authenticated in 2014 by STR testing and no anomalies were detected*
Mycoplasma contamination	*Both cell lines tested negative for mycoplasma contamination.*
Commonly misidentified lines (See ICLAC register)	*HepG2 cell line was used for some experiments because it expresses MAT1A.*

Palaeontology and Archaeology

Specimen provenance	*Provide provenance information for specimens and describe permits that were obtained for the work (including the name of the issuing authority, the date of issue, and any identifying information).*
Specimen deposition	*Indicate where the specimens have been deposited to permit free access by other researchers.*
Dating methods	*If new dates are provided, describe how they were obtained (e.g. collection, storage, sample pretreatment and measurement), where they were obtained (i.e. lab name), the calibration program and the protocol for quality assurance OR state that no new dates are provided.*

☐ Tick this box to confirm that the raw and calibrated dates are available in the paper or in Supplementary Information.

Ethics oversight	*Identify the organization(s) that approved or provided guidance on the study protocol, OR state that no ethical approval or guidance was required and explain why not.*

Note that full information on the approval of the study protocol must also be provided in the manuscript.

Animals and other organisms

Policy information about studies involving animals; ARRIVE guidelines recommended for reporting animal research

Laboratory animals	4-month-old female C57BL/6 mice were purchased from Jacks laboratories and designated as wild-type (WT) mice in this study. All mice are housed under 12-hour light/12-hour dark cycle at an average temperature of 74F and 40% humidity.
Wild animals	*No wild animals were used in this study*
Field-collected samples	*No field-collected samples used in this study*
Ethics oversight	*All procedure protocols, use, and the care of the animals were reviewed and approved by the Institutional Animal Care and Use Committee at Cedars-Sinai Medical Center.*

Note that full information on the approval of the study protocol must also be provided in the manuscript.

Human research participants

Policy information about studies involving human research participants

Population characteristics	All patients had history of alcohol consumption averaging at least 80 g/day for men or 50 g/day for women, for at least 10 years. This criterion is based on epidemiological evidence of the alcohol consumption and cirrhosis relationship. We excluded patients with hepatitis B or C, autoimmune liver disease, hemochromatosis, Wilson disease, and HCC. Liver specimens were obtained rom the explant during liver transplantation. Liver samples from controls were from subjects with no known history of excessive alcohol use or underlying chronic liver disease. They were obtained during abdominal surgeries for various causes. There were no direct subject recruitment into the study, therefore, there is no selection bias. We used the previously collected de-identified human liver tissues from patients with alcohol-associated liver disease who underwent liver transplantation. No other laboratory data are available. The collection of liver tissues were performed under the IRB-approved protocol at the Indiana University Purdue University Indianapolis and Johns Hopkins University.
Recruitment	Written informed consent was obtained from each participant.
Ethics oversight	All liver samples were obtained under a protocol approved by the Human Subjects and Institutional Review Board at Indiana University Indianapolis and Johns Hopkins University.

Clinical data

Policy information about clinical studies

All manuscripts should comply with the ICMJE guidelines for publication of clinical research and a completed CONSORT checklist must be included with all submissions.

Clinical trial registration	*Provide the trial registration number from ClinicalTrials.gov or an equivalent agency.*
Study protocol	*Note where the full trial protocol can be accessed OR if not available, explain why.*
Data collection	*Describe the settings and locales of data collection, noting the time periods of recruitment and data collection.*
Outcomes	*Describe how you pre-defined primary and secondary outcome measures and how you assessed these measures.*

Dual use research of concern

Policy information about dual use research of concern

Hazards

Could the accidental, deliberate or reckless misuse of agents or technologies generated in the work, or the application of information presented in the manuscript, pose a threat to:

No	Yes	
☑	☐	Public health
☑	☐	National security
☑	☐	Crops and/or livestock
☑	☐	Ecosystems
☑	☐	Any other significant area

Experiments of concern

Does the work involve any of these experiments of concern:

No	Yes	
☑	☐	Demonstrate how to render a vaccine ineffective
☑	☐	Confer resistance to therapeutically useful antibiotics or antiviral agents
☑	☐	Enhance the virulence of a pathogen or render a nonpathogen virulent
☑	☐	Increase transmissibility of a pathogen
☑	☐	Alter the host range of a pathogen
☑	☐	Enable evasion of diagnostic/detection modalities
☑	☐	Enable the weaponization of a biological agent or toxin
☑	☐	Any other potentially harmful combination of experiments and agents

ChIP-seq

Data deposition

☐ Confirm that both raw and final processed data have been deposited in a public database such as GEO.

☐ Confirm that you have deposited or provided access to graph files (e.g. BED files) for the called peaks.

Data access links<br>*May remain private before publication.*	*For "Initial submission" or "Revised version" documents, provide reviewer access links. For your "Final submission" document, provide a link to the deposited data.*
Files in database submission	*Provide a list of all files available in the database submission.*
Genome browser session<br>(e.g. UCSC)	*Provide a link to an anonymized genome browser session for "Initial submission" and "Revised version" documents only, to enable peer review. Write "no longer applicable" for "Final submission" documents.*

Methodology

Replicates	*Describe the experimental replicates, specifying number, type and replicate agreement.*
Sequencing depth	*Describe the sequencing depth for each experiment, providing the total number of reads, uniquely mapped reads, length of reads and whether they were paired- or single-end.*
Antibodies	*Describe the antibodies used for the ChIP-seq experiments; as applicable, provide supplier name, catalog number, clone name, and lot number.*
Peak calling parameters	*Specify the command line program and parameters used for read mapping and peak calling, including the ChIP, control and index files used.*
Data quality	*Describe the methods used to ensure data quality in full detail, including how many peaks are at FDR 5% and above 5-fold enrichment.*
Software	*Describe the software used to collect and analyze the ChIP-seq data. For custom code that has been deposited into a community repository, provide accession details.*

Flow Cytometry

Plots

Confirm that:

- [ ] The axis labels state the marker and fluorochrome used (e.g. CD4-FITC).
- [ ] The axis scales are clearly visible. Include numbers along axes only for bottom left plot of group (a 'group' is an analysis of identical markers).
- [ ] All plots are contour plots with outliers or pseudocolor plots.
- [ ] A numerical value for number of cells or percentage (with statistics) is provided.

Methodology

Sample preparation	*Describe the sample preparation, detailing the biological source of the cells and any tissue processing steps used.*
Instrument	*Identify the instrument used for data collection, specifying make and model number.*
Software	*Describe the software used to collect and analyze the flow cytometry data. For custom code that has been deposited into a community repository, provide accession details.*
Cell population abundance	*Describe the abundance of the relevant cell populations within post-sort fractions, providing details on the purity of the samples and how it was determined.*
Gating strategy	*Describe the gating strategy used for all relevant experiments, specifying the preliminary FSC/SSC gates of the starting cell population, indicating where boundaries between "positive" and "negative" staining cell populations are defined.*

- [ ] Tick this box to confirm that a figure exemplifying the gating strategy is provided in the Supplementary Information.

Magnetic resonance imaging

Experimental design

Design type	*Indicate task or resting state; event-related or block design.*
Design specifications	*Specify the number of blocks, trials or experimental units per session and/or subject, and specify the length of each trial or block (if trials are blocked) and interval between trials.*
Behavioral performance measures	*State number and/or type of variables recorded (e.g. correct button press, response time) and what statistics were used to establish that the subjects were performing the task as expected (e.g. mean, range, and/or standard deviation across subjects).*

Acquisition

Imaging type(s)	*Specify: functional, structural, diffusion, perfusion.*
Field strength	*Specify in Tesla*
Sequence & imaging parameters	*Specify the pulse sequence type (gradient echo, spin echo, etc.), imaging type (EPI, spiral, etc.), field of view, matrix size, slice thickness, orientation and TE/TR/flip angle.*
Area of acquisition	*State whether a whole brain scan was used OR define the area of acquisition, describing how the region was determined.*

Diffusion MRI ☐ Used ☐ Not used

Preprocessing

Preprocessing software	*Provide detail on software version and revision number and on specific parameters (model/functions, brain extraction, segmentation, smoothing kernel size, etc.).*
Normalization	*If data were normalized/standardized, describe the approach(es): specify linear or non-linear and define image types used for transformation OR indicate that data were not normalized and explain rationale for lack of normalization.*
Normalization template	*Describe the template used for normalization/transformation, specifying subject space or group standardized space (e.g. original Talairach, MNI305, ICBM152) OR indicate that the data were not normalized.*
Noise and artifact removal	*Describe your procedure(s) for artifact and structured noise removal, specifying motion parameters, tissue signals and physiological signals (heart rate, respiration).*

Volume censoring | *Define your software and/or method and criteria for volume censoring, and state the extent of such censoring.*

Statistical modeling & inference

Model type and settings | *Specify type (mass univariate, multivariate, RSA, predictive, etc.) and describe essential details of the model at the first and second levels (e.g. fixed, random or mixed effects; drift or auto-correlation).*

Effect(s) tested | *Define precise effect in terms of the task or stimulus conditions instead of psychological concepts and indicate whether ANOVA or factorial designs were used.*

Specify type of analysis: ☐ Whole brain ☐ ROI-based ☐ Both

Statistic type for inference
(See Eklund et al. 2016) | *Specify voxel-wise or cluster-wise and report all relevant parameters for cluster-wise methods.*

Correction | *Describe the type of correction and how it is obtained for multiple comparisons (e.g. FWE, FDR, permutation or Monte Carlo).*

Models & analysis

n/a	Involved in the study
☐	☐ Functional and/or effective connectivity
☐	☐ Graph analysis
☐	☐ Multivariate modeling or predictive analysis

Functional and/or effective connectivity | *Report the measures of dependence used and the model details (e.g. Pearson correlation, partial correlation, mutual information).*

Graph analysis | *Report the dependent variable and connectivity measure, specifying weighted graph or binarized graph, subject- or group-level, and the global and/or node summaries used (e.g. clustering coefficient, efficiency, etc.).*

Multivariate modeling and predictive analysis | *Specify independent variables, features extraction and dimension reduction, model, training and evaluation metrics.*
